# Fabrication and characterization of PHEMA–gelatin scaffold enriched with graphene oxide for bone tissue engineering

**DOI:** 10.1186/s13018-022-03122-4

**Published:** 2022-04-09

**Authors:** Sara Tabatabaee, Nafiseh Baheiraei, Mojdeh Salehnia

**Affiliations:** 1 Department of Bio-Computing, Faculty of Interdisciplinary Science and Technology, Tarbiat Modares University, Tehran, Iran; 2 Tissue Engineering and Applied Cell Sciences Division, Department of Anatomical Sciences, Faculty of Medical Sciences, Tarbiat Modares University, Tehran, Iran; 3 Department of Anatomical Sciences, School of Medical Sciences, Tarbiat Modares University, Tehran, Iran

**Keywords:** Bone tissue engineering, Scaffold, PHEMA, Graphene oxide

## Abstract

**Background:**

Growing investigations demonstrate that graphene oxide (GO) has an undeniable impact on repairing damaged bone tissue. Moreover, it has been stated in the literatures that poly(2-hydroxyethyl methacrylate) (PHEMA) and gelatin could provide a biocompatible structure.

**Methods:**

In this research, we fabricated a scaffold using freeze-drying method comprised of PHEMA and gelatin, combined with GO. The validation of the successful fabrication of the scaffolds was performed utilizing Fourier-transform infrared spectroscopy (FTIR) and X-ray diffraction assay (XRD). The microstructure of the scaffolds was observed using scanning electron microscopy (SEM). The structural properties of the scaffolds including mechanical strength, hydrophilicity, electrical conductivity, and degradation rate were also evaluated. Human bone marrow‐derived mesenchymal stem cells (hBM-MSCs) were used to evaluate the cytotoxicity of the prepared scaffolds. The osteogenic potential of the GO-containing scaffolds was studied by measuring the alkaline phosphatase (ALP) activity after 7, 14, and 21 days cell culturing.

**Results:**

SEM assay showed a porous interconnected scaffold with approximate pore size of 50–300 μm, appropriate for bone regeneration. The increase in GO concentration from 0.25 to 0.75% w/v exhibited a significant improvement in scaffolds compressive modulus from 9.03 ± 0.36 to 42.82 ± 1.63 MPa. Conventional four-probe analysis confirmed the electrical conductivity of the scaffolds in the semiconductor range. The degradation rate of the samples appeared to be in compliance with bone healing process. The scaffolds exhibited no cytotoxicity using MTT assay against hBM-MSCs. ALP analysis indicated that the PHEMA–Gel–GO scaffolds could efficiently cause the differentiation of hBM-MSCs into osteoblasts after 21 days, even without the addition of the osteogenic differentiation medium.

**Conclusion:**

Based on the results of this research, it can be stated that the PHEMA–Gel–GO composition is a promising platform for bone tissue engineering.

## Background

Bone defects are a major public health concern and the leading cause of disability and morbidity in the elderly patients [1]. Although the existing therapies have been quite successful, they are correlated with considerable limitations. Autografting, the gold standard for bone treatment, with its remarkable outcomes, could be restricted by important complications; such as limitations in availability, donor site morbidity, pain, and infection [2, 3]. Although allografting influences the osteoconductivity as well as the capability of application in large and differently shaped pieces of bone defects, it could be accompanied by the risk of potential infection, nonunion fatigue fracture, disease transmission, defective biomechanical and biochemical properties in comparison with the native tissue and rejection [3–5]. Although metallic- and ceramic- based artificial implants may be considered as immediate solutions for supporting the damaged sites, they are limited by issues like corrosion failure, low tensile strength, brittleness, and the possibility of poor integration with the surrounding tissue [3]. Therefore, there is an essential need to optimize the existing treatment methods for bone-related disorders [6].

Bone tissue engineering, as an alternative treatment strategy, has provided novel methods for substituting the disordered or damaged bone leading to the tissue regeneration by utilizing different osteogenic cells, temporary scaffolds, and growth factors [7]. For the aim of achieving the optimum results in healing, the scientists in bone tissue engineering have attempted to design scaffolds with a bone-like structure and chemical compositions [8, 9]. Natural or synthetic biocompatible materials have been selected for fabricating the scaffolds to mimic the extracellular matrix (ECM) of bone [10]. Specifically, in recent years, various synthetic polymers such as polylactic acid (PLA) [11], polyglycolic acid (PGA) [12], and polycaprolactone (PCL) [13] have been employed to accomplish the aforementioned requirements based on biomimetic strategies. Among these materials, PHEMA has gained attentions due to its significant biocompatibility and physicochemical properties, resembling those of living tissues [14]. PHEMA is a flexible and stable synthetic polymer composed of 2-hydroxyethyl methacrylate monomer units, which does not require a complicated fabrication process. Interestingly, many of physical characteristics of this polymer such as rheology, hydrophilicity, degradability, hardness, and swelling in case of water inflow could be regulated by techniques such as cross-linking or copolymerization. PHEMA has been broadly used in the forms of hydrogels, cryogels, and implant coatings for biomedical applications such as cardiac, bone, and cartilage tissue engineering, reconstruction of spinal cord injuries and replacement of lumbar disks, and drug-delivery systems [15, 16]. In particular, researchers have obtained remarkable results using PHEMA in combination with various types of materials for bone tissue engineering in recent investigations [17–20]. Shahrousvand et al. fabricated a porous scaffold composed of PHEMA, polyurethane, and cellulose nanowhiskers seeded by hBM-MSCs for bone tissue engineering. Based on the obtained results, the authors considered the scaffold as an appropriate composition for bone scaffold due to its efficient impact on cell proliferation, bone mineralization, and capability of osteogenic differentiation [21].

Despite noting advantages mentioned above, the surface of PHEMA does not facilitate cell adhesion and proliferation. This issue creates constraints in biological circumstances when cell adhesion is required to progress [22]. A wide range of natural and synthetic polymers have been utilized for overcoming this problem [23–29]. Among natural polymers, gelatin (Gel) is a biodegradable and biocompatible polymer derived from thermal and chemical denaturation of collagen, comprised of arginyl–glycyl–aspartic acid (RGD)-like sequence which enhances cell adhesion, proliferation, and migration [27, 28]. Gel has led to desirable results in recent bone tissue engineering investigations [27, 30, 31]. Cetin et al. examined the osteogenic activity of a super porous hydrogel of PHEMA and Gel seeded by preosteoblastic MC3T3-E1 cells. In the presence of Gel, mechanical strength and elastic modulus were improved, as well as cell attachment, proliferation, and differentiation. Therefore, the authors considered PHEMA–Gel composition as a potential scaffold for bone tissue engineering [23].

Due to the piezoelectric characteristic of some tissues, notably bone tissue, electrically active materials have recently received a great deal of interest in the field of tissue engineering [32]. These materials with their tailorable chemical, physical, and electrical properties are able to mimic the bone-like structure at cellular and tissue levels. Electroactive materials have remarkable effect on the ultimate fate of cells and tissues via improving the adhesion of the biological molecules and facilitating intracellular pathways [8, 33–35]. Among electroactive moieties, carbon-based nanomaterials have been widely used as reinforcing agents for biomedical engineering investigations [36–38]. GO, for example, is fabricated by oxidation of graphite, and has demonstrated a significant potential to be applied as a modifier in the composition of scaffolds. Reorganization of the cell cytoskeleton has a substantial influence on increasing cellular processes such as cell adhesion, proliferation, and differentiation [39]. Compared with other carbon-based nanomaterials, high mechanical stiffness as well as its bioactivity, make GO an excellent candidate for bone tissue engineering [40, 41]. In a study by Hermenean et al., enhanced cell attachment, proliferation, and differentiation as well as biomimetic mineralization and promoted bone regeneration in mouse calvarial defects were observed in a scaffold composed of chitosan functionalized with GO for bone applications [42]. In another research, combination of GO with alginate and Gel presented more compressive strength and hydrophilicity, appropriate biodegradation, and upregulation of cellular functions such as differentiation for bone regeneration compared to the pure Gel–alginate scaffold [27].

Here, in this study, our goal was to fabricate a porous scaffold composed of PHEMA, Gel, and GO via freeze-drying method to benefit from the biocompatibility of PHEMA and Gel and the electroactivity of GO. We hypothesized that the incorporation of GO into the structure of PHMEA and Gel could enhance the hBM-MSCs functions by improving cell–cell interactions. Also, the physicochemical characteristics of the scaffolds were assessed by the appropriate evaluations. To the best of our knowledge, the present research is the first investigation on a bone scaffold comprised of PHEMA, Gel, and GO.

## Methods

### Polymer synthesis

PHEMA was fabricated using radical polymerization according to the previous methods [43]. First, 10 mL of toluene (Sigma) was poured in the flask as the solvent. Then, 0.1 g (0.6 mmol) of AIBN (Sigma) as the radical initiator and 5 mL (38.4 mmol) of the HEMA monomer (Sigma) was added to the solvent. The mixture was placed in the oil bath at 70 °C and stirred gently. Nitrogen purging was employed for the first 10 min to displace any undesirable atmosphere with an inert nitrogen atmosphere. The reaction mixture was maintained at the mentioned circumstances for 24 h. The final product was then dried in freeze-dryer device (Alpha 2-4 LDplus, Martin Christ) to remove any residual solvent.

### Preparation of the scaffolds

Scaffolds were fabricated using freeze-drying method. The homogeneous solution containing synthesized PHEMA in dimethylformamide (DMF; Sigma) was added to the aqueous solution of Gel (Sigma) followed by dropwise addition of different GO (GrapheneX, Kimia Pishtaz) concentrations in DMF solution. Total concentration of both PHEMA and Gel was determined 4% w/v. The obtained mixture was cast in Teflon molds and froze at temperature of -20 °C for 8 h and then − 80 °C for 12 h. Samples were then lyophilized in freeze-dryer device (Alpha 2-4 LDplus, Martin Christ) for 48 h at − 80 °C. After that, a 25 mM of 1-ethyl-3-(3-dimethylaminopropyl) carbodiimide (EDC; Merck) and 25 mM of *N*-hydroxysuccinimide (NHS; Merck) solution was used to cross link the scaffolds for 24 h. Samples were then washed several times by deionized (DI) water before being lyophilized again for 24 h.

### Scaffold characterizations

To investigate the chemical composition and bonds of the samples, FTIR (PerkinElmer, Frontier) was utilized in a range of 500–4000 cm^−1^, with a resolution of 1 cm^−1^, at a scan speed of 32 scans/min, in KBr-diluted medium. The crystalline phase of fabricated scaffolds was analyzed using XRD (X’Pert MPD, Philips) with Cu anode at a fixed incident angle of 0.02 in a 2θ range of 5–100°. The morphology and microstructure of the scaffolds were also evaluated by SEM (XL30, Philips) after gold coating. The image j software was used for measuring the pore size according to the previous literatures [44, 45]. To study the mechanical properties of the scaffolds, compression strength test was performed using a universal testing machine (H10KS; Hounsfield) with a 25 kN load cell and a loading rate of 1 mm/min. Compressive modulus of the scaffolds was reported based on the slope of the stress–strain curve in the linear region. Surface wettability of the scaffolds was investigated by the water contact angle assessment via an optical video contact angle system (OCA-15-plus, Dataphysics) using the sessile drop method for at least three different spots of each scaffold. After 4 μl water droplets were poured on the surface of the scaffolds, shape changes were recorded and the surface contact angles were measured. The electrical conductivity of the scaffolds was assessed by the four-point probe method (196 system DMM, Keithley). The electrical current was obtained after placing each sample on the apparatus and applying voltage. The scaffolds electrical conductivity was reported using the following formula:

1 $$\sigma = \, \left( {{2}.{44 } \times { 1}0 \, /S} \right) \, \times \, \left( {I/E} \right)$$

where *σ* is the electrical conductivity in siemens per meter (S/m), *S* is the side area of the sample in m^2^, *I* is the electrical current through the outer probe in amp, and *E* is the voltage drop across the inner probe in V. Also, 2.44 is referred to the systematic constant.

Bioactivity of the prepared scaffolds was studied by being soaked in simulated body fluid (SBF) at 37 °C after 7 days Followed by washing with DI water and drying. The morphology and the ratio of Ca/P crystals formed on the surface of the scaffolds were examined by SEM equipped with an energy-dispersive X-ray analyzer (EDX; Rontec).

For investigating the scaffolds swelling behavior, the samples with certain dry weights were immersed in phosphate-buffered saline (PBS) (pH = 7.4) solution at 37 °C for 0.5, 1, 3, 6, 24, 26, 48, and 50 h. At each time point, the scaffolds were taken out, the surface water was removed using a filter paper, and their wet weight was recorded. The swelling ratio was calculated by the following equation:

2 $${\text{Swelling}}\,{\text{ratio}}\,\left( \% \right) = \left( {{\text{wet}}\,{\text{weight}} - {\text{dry}}\,{\text{weight}}/{\text{dry}}\,{\text{weight}}} \right) \, \times { 1}00$$

For evaluating the hydrolytic degradation of the scaffolds, prior to immersing in PBS (pH = 7.4) at 37 °C, the weight of dried and sterilized samples was measured. Then at certain time points, the scaffolds were removed, washed with deionized water, and dried in a vacuum. The percentage of weight loss was calculated by the following formula:

3 $${\text{Weight}}\,{\text{loss}}\,\left( \% \right) = \left( {W_{1} - W_{2} } \right)/W_{1} \times { 1}00$$

where *W*_1_ and *W*_2_ are referred to the weights of the scaffold before and after degradation, respectively. Also, after removing the scaffolds at each time point, the pH changes of the PBS solutions were recorded.

### Cellular evaluations

#### Cytotoxicity and morphology investigations

In this study, hBM-MSCs (Royan Stem Cell Bank, Royan Institute) were used to evaluate the cytotoxicity of the prepared scaffolds. Cells were cultured in the Dulbecco’s modified Eagle medium F12 (DMEMF12; Invitrogen) medium supplemented with 10% (v/v) fetal bovine serum (FBS, Gibco) and 1% antibiotic penicillin/streptomycin (Sigma). Prior to the cell culture, scaffolds were sterilized by ethanol 70% v/v, rinsed in PBS and placed under ultraviolet (UV) radiation for 15 min each side. Then, 8 × 10^3^ cells were seeded over the scaffolds in 96-well culture plates, and incubated in 5% CO_2_ at 37 °C. After 48, cytocompatibility assessment was carried out by MTT (Sigma) colorimetric assay. Briefly, the culture medium was removed, and 100 μL MTT solution (5 mg/mL in PBS) was poured into each well followed by incubation of the samples for 4 h. Then, the medium was removed and the formazan crystals were dissolved using dimethyl sulfoxide (DMSO; Sigma). The optical absorbance was evaluated with a microplate reader (ELISA reader; ELX808, BioTek) at 540 nm. Cells cultured on tissue culture plate (TCP) were considered as the control group. Also, the morphology of the cultured hBM-MSCs on the fabricated scaffolds was assessed by SEM. after 48 h, cells were fixed using 2.5% glutaraldehyde (GA; Sigma) for one hour followed by washing in PBS for several times. Samples were then dehydrated with graded ethanol series (30%, 70%, 90%, and 100%), and coated with gold under vacuum to be prepared for SEM evaluation.

#### Alkaline phosphatase activity

The osteogenic potential of the GO-contained scaffolds was examined by measuring the alkaline phosphatase activity of the hBM-MSCs being cultured on the scaffolds after 7, 14, and 21 days. At first, cells were washed with PBS (pH = 7.4) and homogenized in 1 mL assay buffer using sonication. Then, a mixture of 0.1 mL of the cell lysate and 0.2 mL of p‐nitrophenylphosphate (pNPP) substrate solution (BioVision) was prepared, and incubated for 30 min at 37 °C. After that, 2 M NaOH solution was added for ending the reaction. The absorbance was reported by a microplate reader (Stat Fax 3200; Awareness Technology) at 405 nm. Also, the normalization of the ALP quantity in the cultured cells was performed against the total protein content. The negative and positive control groups were considered cells cultured on TCP with and without osteogenic differentiation medium, respectively.

### Statistical analysis

All results were reported as mean ± standard deviation (SDs). The data comparison was carried out by one-way analysis of variance (ANOVA) employing SPSS 16.0 software (SPSS) after ensuring of the normality as well as the homoscedasticity of the results. At least three samples were examined for each experiment. Each experiment was run at least 3 times and in case of obtaining the similar results, it was statistically analyzed and reported as the final result. The differences with *p* values of < 0.05 were considered to be significant.

## Results and discussion

An ideal bone scaffold should meet several criteria to be introduced as an appropriate alternative of the other therapeutic approaches. Based on the previous investigations, although PHEMA–Gel scaffold presented significant biological responses, it does not have adequate mechanical strength which is an essential factor for a load-bearing tissue such as bone [23]. It has been proved that the presence of GO could enhance the scaffold structural strength [27]. Also, it is important for a scaffold to have a degradation rate in compliance with the healing process of the tissue. It is demonstrated that GO could improve the degradation rate of the scaffold to an acceptable extent [27]. On the other hand, GO as a carbon-based nanomaterial can mimic the electrophysiologic environment of bone tissue by its electroactive characteristic [46]. Therefore, the aim for selecting these components was to benefit from each one of the materials advantages such as the stability of PHEMA, the cellular interaction of Gel, and the electrical conductivity, the mechanical strength, and the osteogenic activity of GO. All together could result in fabricating a scaffold with optimum structural and cellular properties for bone tissue engineering.

### Scaffold characterizations

**Fig. 1:**
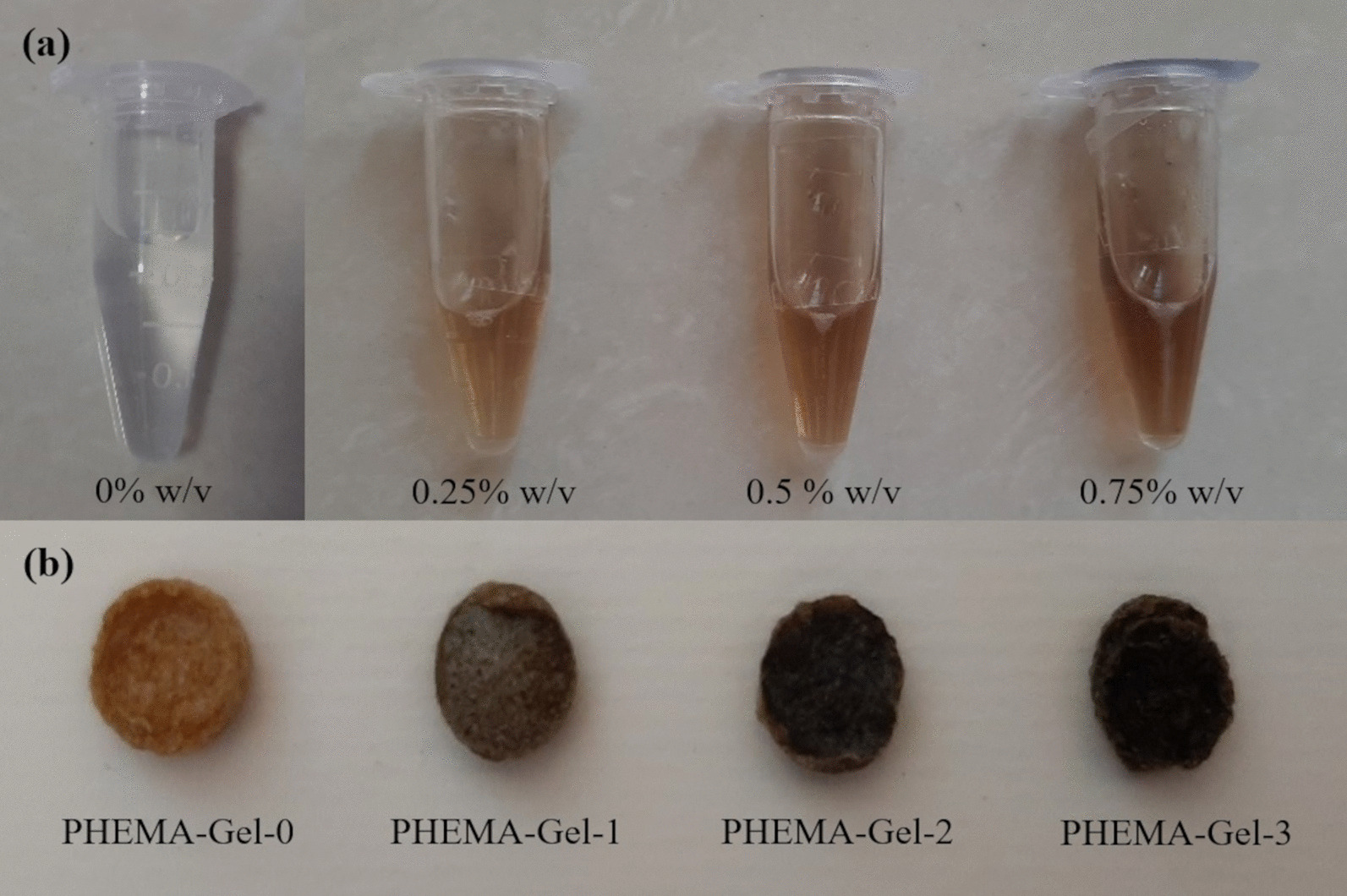
**a** GO solutions in DMF. **b** Microscopic images of the scaffold with different concentrations of GO

The prepared scaffolds were appointed as shown in Table 1. Also, the obtained GO solutions in DMF with different concentrations and the microscopic images of the prepared scaffolds by freeze-drying method are displayed in Fig. 1.

**Table 1:** Scaffold preparations and groups

Abbreviations	Scaffolds
PHEMA–Gel-0	PHEMA + Gel
PHEMA–Gel-1	PHEMA + Gel + 0.25% w/v GO
PHEMA–Gel-2	PHEMA + Gel + 0.5% w/v GO
PHEMA–Gel-3	PHEMA + Gel + 0.75% w/v GO

**Fig. 2:**
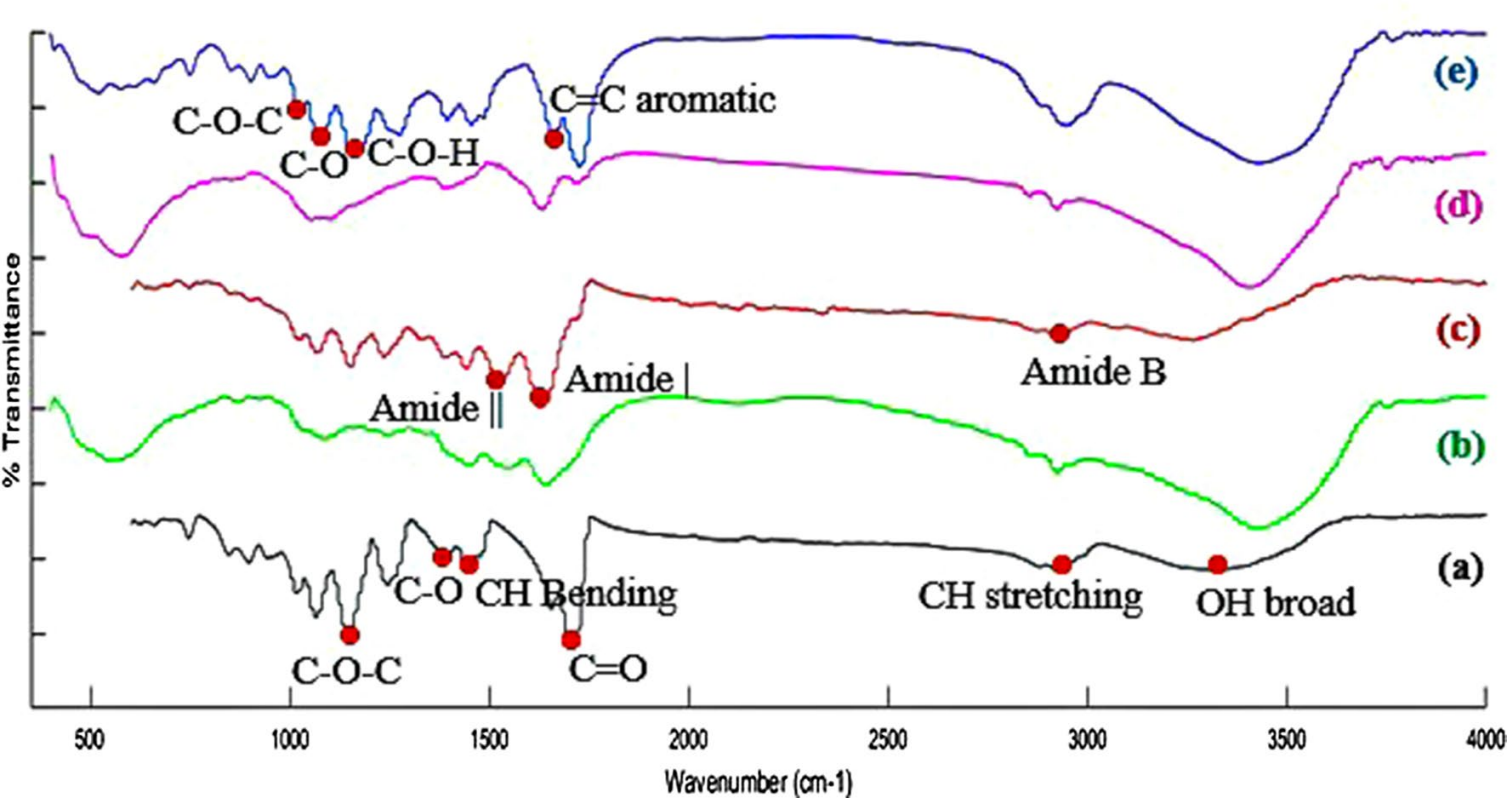
FTIR spectra of (a) PHEMA, (b) Gel, (c) PHEMA–Gel scaffold, (d) GO, and (e) PHEMA–Gel–GO scaffold

Figure 2 shows the results of the FTIR spectra of the synthesized samples as well as GO powder. In the spectrum of PHEMA (Fig. 2-a) the O–H broad peak in the range of 3220–3390 cm^−1^, C–H stretching at 2940 cm^−1^, C=O stretching at 1710 cm^−1^, C–H bending at 1450 cm^−1^, C–O stretching at 1380 cm^−1^, and C–O–C stretching at 1150 cm^−1^ could be attributed to this polymer [47–49]. The detected peaks in the spectrum of Gel (Fig. 2b) are associated with O–H band at 3430 cm^−1^, amide B at 2930 cm^−1^, amide I at 1650 cm^−1^, and amide II at 1540 cm^−1^ [34]. In the spectrum of PHEMA–Gel (Fig. 2c), the characteristic peaks of PHEMA are O–H peak at 3420 cm^−1^, C–H stretching at 2880 cm^−1^, C=O stretching at 1630 cm^−1^, C–H bending at 1440 cm^−1^, C–O stretching at 1390 cm^−1^, and C–O–C stretching at 1150 cm^−1^ [47–49]. The mentioned O–H peak at 3420 cm^−1^, could also be related to Gel as well as, amide B peak at 2930^–1^ and amide II peak at 1530 cm^−1^. It should be stated that in this spectrum, the intense peak at 1630 cm^−1^ can be attributed to both carbonyl groups of PHEMA and amide I of Gel [34]. The characteristic peaks in spectrum of GO (Fig. 2-d) are O–H band at 3420 cm^−1^, C=O at 1720 cm^−1^, C = C aromatic at 1630 cm^−1^, C–O–H at 1390 cm^−1^, C–O stretching at 1150 cm^−1^, and C–O–C at 1060 cm^−1^ [50]. For PHEMA–Gel–GO (Fig. 2-e), the PHEMA-related peaks are O–H peak at 3420 cm^−1^, C–H stretching at 2940 cm^−1^, C=O stretching at 1720 cm^−1^, C–H bending at 1450 cm^−1^, C–O stretching at 1400 cm^−1^, and C–O–C stretching at 1160 cm^−1^ [47–49]. Moreover, the visible peaks of Gel in the spectrum are O–H band at 3420 cm^−1^, amide B at 2940 cm^−1^, amide I at 1660 cm^−1^, and amide II at 1480 cm^−1^ [34]. Also, the absorption of GO functional groups corresponds to the intense peak of O–H at 3420 cm^−1^, C=O at 1720 cm^−1^, C = C aromatic at 1660 cm^−1^, C–O–H at 1390 cm^−1^, C–O at 1160 cm^−1^, and C–O–C at 1070 cm^−1^ [50]. Based on the data obtained from FTIR spectra, the synthesis of PHEMA via radical polymerization was successfully accomplished and the fabricating of the scaffolds composed of PHEMA–Gel as well as, PHEMA–Gel–GO was approved.

**Fig. 3:**
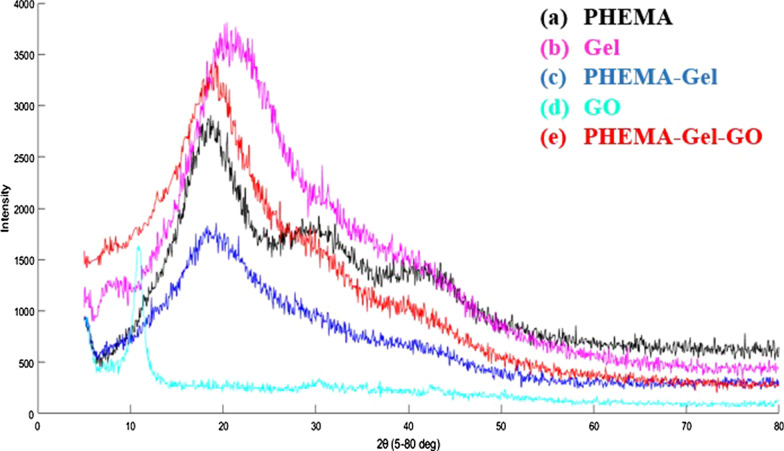
XRD patterns of (a) PHEMA, (b) Gel, (c) PHEMA–Gel scaffold, (d) GO, and (e) PHEMA–Gel–GO scaffold

Figure 3 presents the XRD patterns of the prepared scaffolds in the angle range of 5° < 2*θ* < 80°. In the pattern of pure PHEMA (Fig. 3-a), a relatively broad peak can be observed at 19.5°. Also, two quite broad peaks are shown at 30° and 41.3° which reveal the amorphous nature of PHEMA [51]. Figure 3-b indicates the amorphous structure of pure Gel with a wide peak at 21°, as well [52]. For PHEMA–Gel scaffolds (Fig. 3-c), a wider peak in the area of 19° with less intensity compared to pure PHEMA and pure Gel as well as a minor peak at 40° are visible. The results suggest that the structure of PHEMA–Gel composite has even less crystallinity than its individual components [51, 52]. A peak at 10.9° is associated with the lattice *d*-spacing of 0.82 nm of GO in Fig. 3-d [53]. In the XRD pattern of PHEMA–Gel–GO scaffold (Fig. 3-e), the presence of GO improved the crystallinity of the composite scaffold based on the observation of a sharper peak at 18.5° and the broad peaks at 30° and 40° related to PHEMA [46, 51, 52]. The absence of regular GO diffraction can be related to the homogenous dispersion of GO in the matrix of PHEMA and Gel which causes in the foundation of an exfoliated structure [54].

**Fig. 4:**
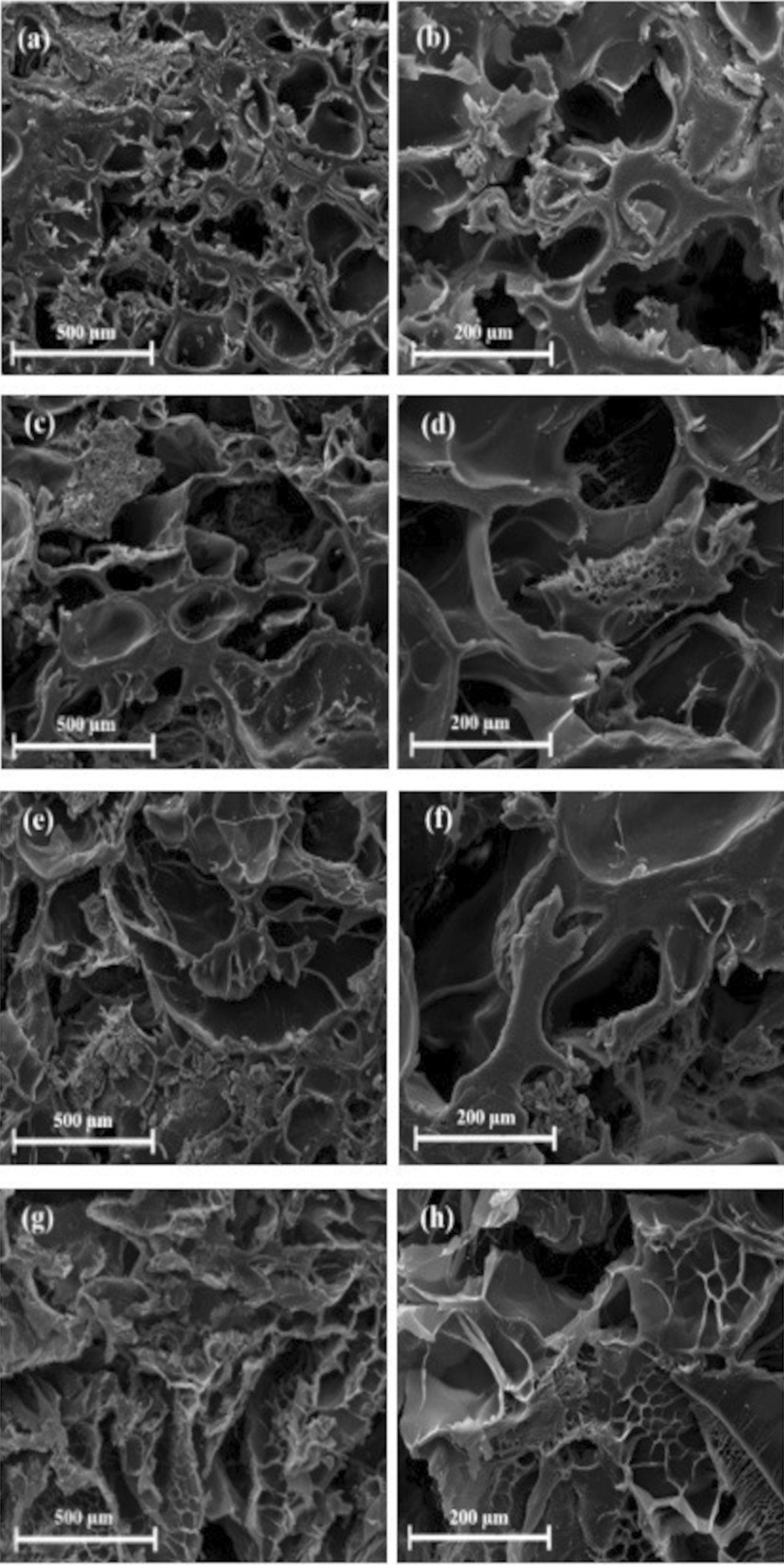
SEM images of the scaffolds with different magnitudes, **a**, **b** PHEMA–Gel-0, **c**, **d** PHEMA–Gel-1, **e**, **f** PHEMA–Gel-2, and **g**, **h** PHEMA–Gel-3

The SEM images of the prepared scaffolds are shown in Fig. 4. Accordingly, it can be stated that the obtained structures were porous and interconnected with random orientations. One of the most essential criteria in determining an ideal scaffold in tissue engineering is the interconnectivity of the pores [4]. The pore size ranges were estimated to be 100 ± 10 to 300 ± 10 μm for PHEMA–Gel-0 and PHEMA–Gel-1 as well as, 50 ± 5 to 200 ± 8 μm for PHEMA–Gel-2 and PHEMA–Gel-3. Based on the previous investigations, it could be stated that the mentioned ranges are appropriate for growth and regeneration of bone tissue [55]. Moreover, according to the SEM images, it seems that an increase in GO concentration led to a decrease in the average pore sizes which has also been confirmed in the earlier researches. In an assessment of the bone scaffolds containing PCL and GO by Mohammadi et al., it was observed that a 0.5% increase in GO clearly reduces the size of the pores [13].

**Fig. 5:**
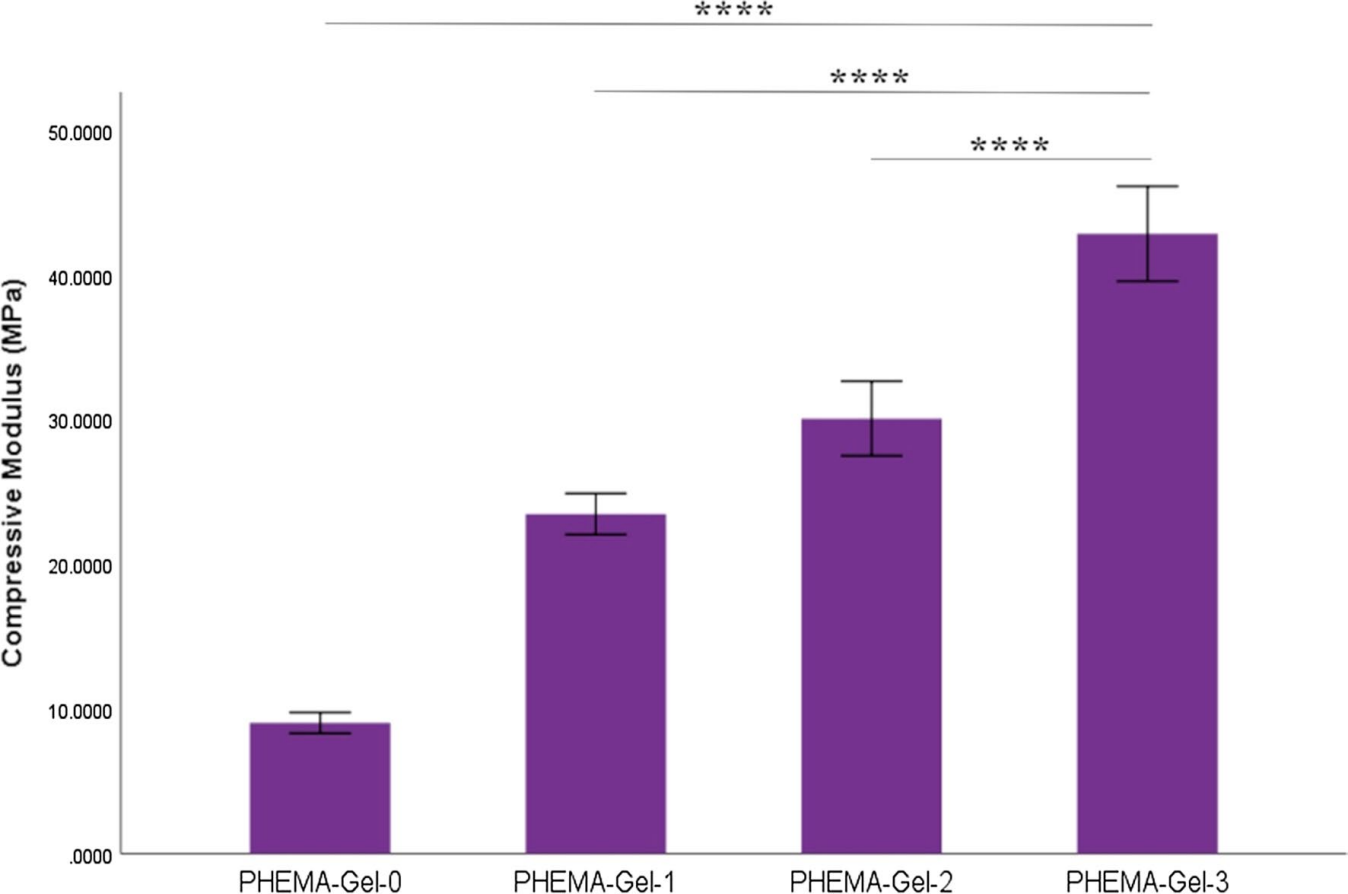
Compressive modulus of the scaffolds (****p value < 0.0001 compared to the other groups)

Mechanical characteristics of the scaffolds present a key role for providing structural support and load-bearing ability to the bone tissue [56]. Furthermore, mechanical properties have an undeniable impact on regulating subcellular, cellular, and tissue responses [13]. Figure 5 displays the compressive modulus (linear region slopes of the stress–strain curves) of the scaffolds bearing the 25 kN load cell with a loading rate of 1 mm/min. As can be seen, the compressive modulus increased with GO concentration from 9.03 ± 0.36 MPa for PHEMA–Gel-0 to 42.82 ± 1.63 MPa for PHEMA–Gel-3 and significant enhancement could be observed between all the samples (*p* value < 0.0001). The obtained results were higher than that was reported by Babic et al.; a scaffold composed of PHEMA, Gel, alginate and GO being fabricated by freeze-drying method [57].

Moreover, considering the enhancement of the compressive strength (as the slope of the stress–strain curve) by GO increase, it can be deduced that increasing the GO concentration enhanced the toughness (ability to absorb energy before failure) of the scaffolds extracted from the area below the stress–strain curve [58]. These improvements could be correlated to the state that GO might act as a crack resistor for preventing the propagation of the cracks [59]. It is well known that the bone tissue should bear different amounts of stress in life time which causes many microcracks; thus, crack resisting is a significant property for a bone scaffold [60]. Due to the ability of GO to establish a relatively strong interaction with the polymer matrices, this interaction could create the bridges between the microcracks which leads to stopping the propagation of the microcracks and preventing the formation of the larger cracks [61]. Therefore, as the concentration of GO increases, the greater distribution of the GO particles occurs and this will establish stronger interactions, make the scaffolds stiffer, and augment the scaffolds compressive strength [62]. The mechanical evaluation results agreed with the previous investigations; Purohit et al. revealed that the compressive strength of a scaffold composed of Gel and alginate could be tailored by varying the GO concentration. The results indicated that the compressive modulus of the scaffold has an improvement of 83% by addition of GO and increasing its concentration to 0.3% w/v [27]. Also, in another research, the compressive strength assessment of a structure comprised of collagen, chitosan, alginate, and GO approved the reinforcing influence of GO on the polymer matrix leading to the enhancement of the scaffold stiffness and compressive modulus [63]. Furthermore, a compressive modulus in the range of 1.5–45 is equivalent to the cancellous bone. This means that compressive strength of the prepared scaffolds is appropriate for applying in cancellous bone [64].

**Fig. 6:**
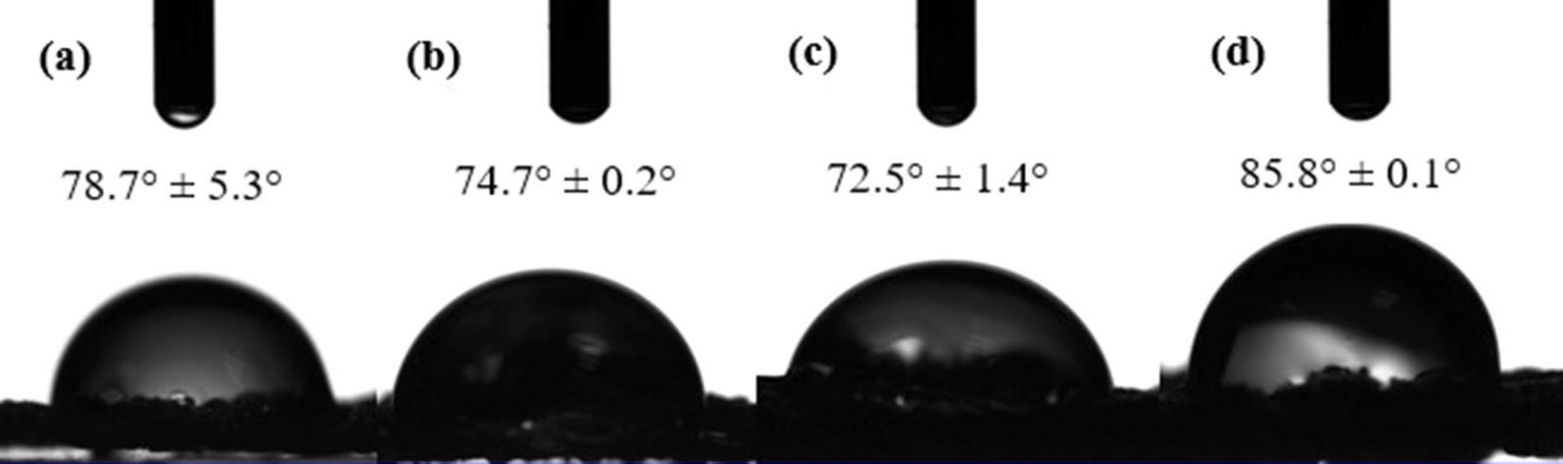
Water contact angle of **a** PHEMA–Gel-0, **b** PHEMA–Gel-1, **c** PHEMA–Gel-2, and **d** PHEMA–Gel-3

An important feature that impacts on the interaction between the cells and the surface of the scaffolds is hydrophilicity which was evaluated by water contact angle assessment. As expected, the contact angle of the scaffolds (Fig. 6) was decreased by increasing the concentration of GO from 78.7° ± 5.3° for PHEMA–Gel-0 to 72.5° ± 1.4° for PHEMA–Gel-2 which could be associated with a large number of the hydrophilic functional groups such as COOH, C=O, O–H, and C–O–C in the structure of GO [8, 27]. The improvement of hydrophilicity via increasing GO concentration has been confirmed in previous researches. It was demonstrated in a study by Zhou et al. that a GO concentration increases from 0 to 1 percent w/v within polyhydroxybutyrate matrix causing a 54º reduction of water contact angle [9]. Aidun et al. reported the enhancement of surface hydrophilicity by increasing the rate of GO in an electrospun composition of PCL, chitosan, and collagen even with the reduction of mean pore size [65]. However, surprisingly, there was an increase in the contact angle of PHEMA–Gel-3 to 85.8° ± 0.14°. The reason might be the existing carbonyl (C=O) and methyl (CH_3_) groups in PHEMA and GO which could lead to the hydrophobicity of the scaffolds [66]. Therefore, with an excessive increase in GO content, the mentioned hydrophobic functional groups may overcome the hydrophilicity of the structure which results in water contact angle increase. Based on the previous researches, it was suggested that the suitable range of water contact angle for the scaffolds to interact with different types of cells is in the range of 40°–80° [67]. Hence, it could be concluded that except for the PHEMA–Gel-3, the synthesized scaffolds have appropriate hydrophilicity for cell interactions.

The electrical activity of the scaffold could affect stimulating bone cells. The results of the electrical conductivity assay are shown in Table 2. The conductivity of the scaffolds is enhanced by the increase in GO concentrations and reached the range of 10^–3^ S/ m for PHEMA–Gel-2 and PHEMA–Gel-3. The results of this test are in accordance with previous researches [46]. Also, an electroactive scaffold composed of GO and chitosan which was fabricated by Jiang et al. demonstrated the optimized electrical conductivity of 1.6 * 10^–3^ S/m [68].

**Table 2:** Electrical conductivity of the prepared scaffolds

Scaffolds	PHEMA–Gel-0	PHEMA–Gel-1	PHEMA–Gel-2	PHEMA–Gel-3
Electrical conductivity (S/m)	4.48 ± 0.16 (* 10^–5^)	3.51 ± 0.05 (* 10^–4^)	1.10 ± 0.04 (* 10^–3^)	1.55 (* 10^–3^)

**Fig. 7:**
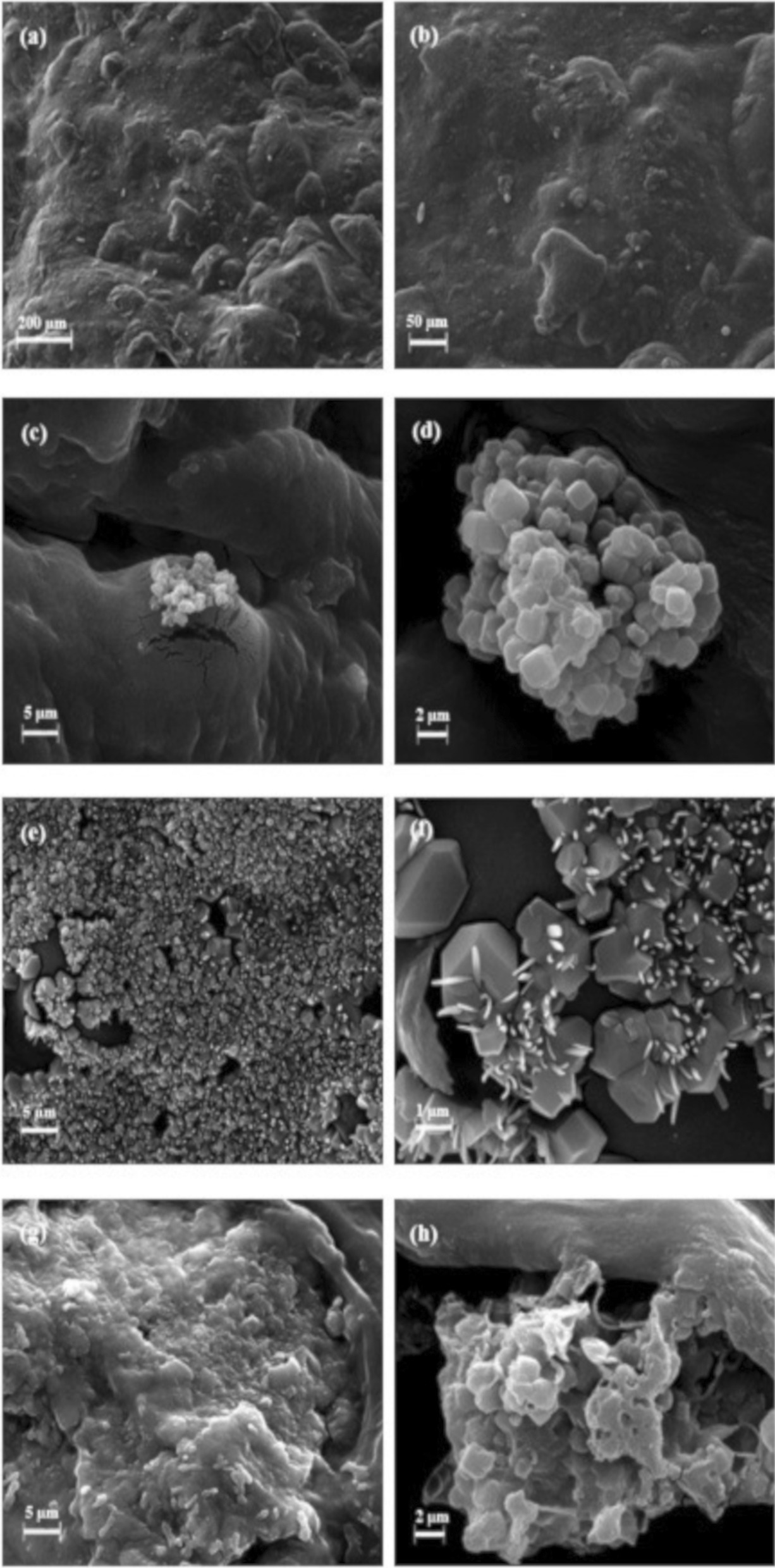
SEM images of the scaffolds immersed in SBF for 7 days with different magnitudes, **a**, **b** PHEMA–Gel-0, **c**, **d** PHEMA–Gel-1, **e**, **f** PHEMA–Gel-2, and **g**, **h** PHEMA–Gel-3

For the aim of evaluating the bioactivity potential of the prepared scaffolds in vivo, samples were immersed in SBF for 7 days and the apatite formation on their surface was investigated. Based on the SEM images, no calcium phosphate (CP) formation was observed on the surface of the PHEMA–Gel-0 (Fig. 7a, b). In PHEMA–Gel-1 (Fig. 7-c and 7-d), the formation of CP crystallites is visible. On the surface of PHEMA–Gel-2 (Fig. 7-e and 7-f), a high amount of scattered fine CP crystals can be remarkably observed with an approximate size of 2 µm [69]. The SEM pictures of PHEMA–Gel-3 (Fig. 7f, g), display the crystallites and the primitive steps of fine CP crystal formation on their surface. According to the SEM results, it could be concluded that the presence of GO in the structure of the scaffolds improves the calcium phosphate formation which could enhance the bone formation potential in vivo. Among the scaffolds studied in this research, PHEMA–Gel-2 and PHEMA–Gel-3 seem to provide an optimized environment appropriate for the bioactivity of the scaffold.

**Fig. 8:**
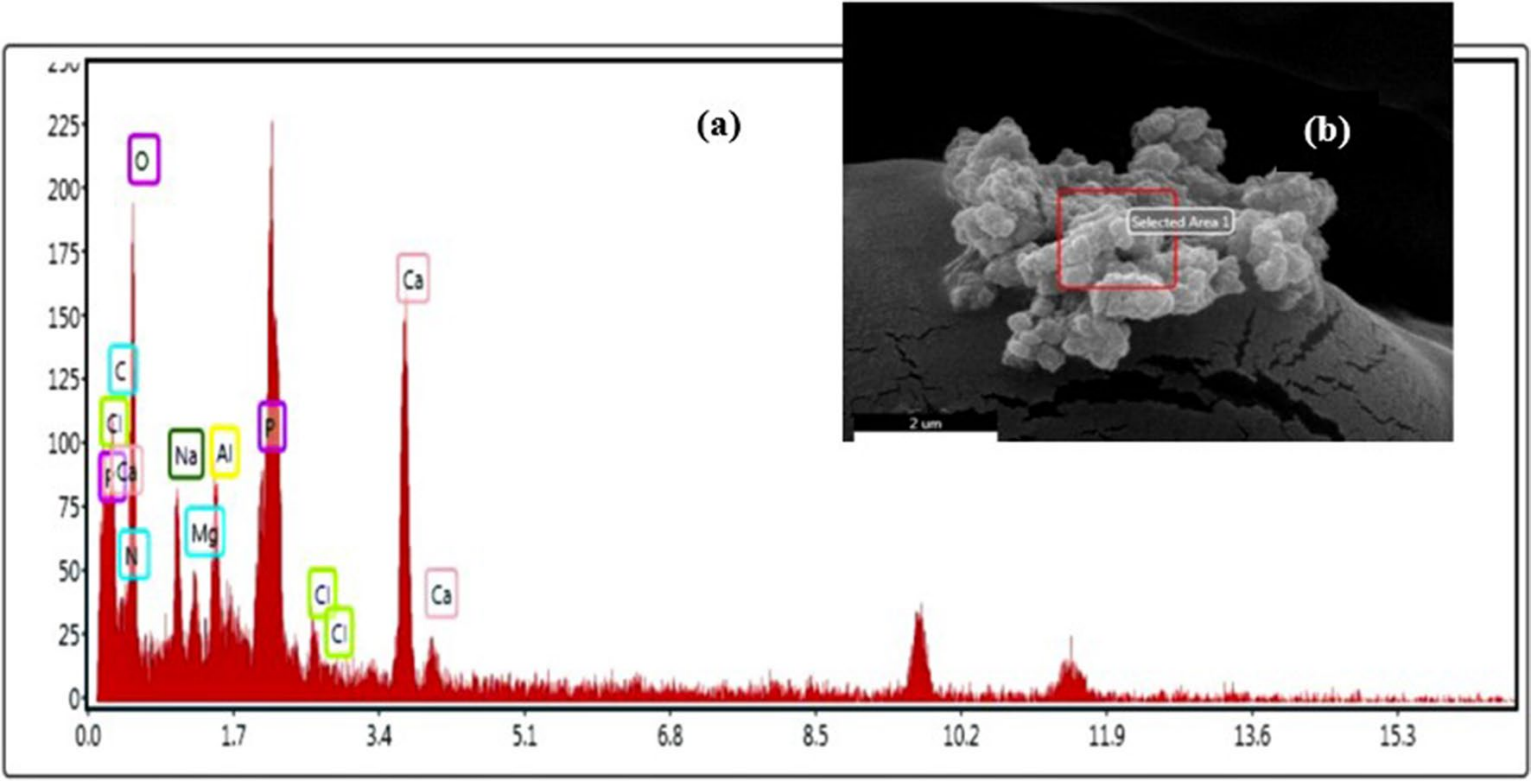
**a** EDS spectra of the PHEMA–Gel–GO scaffold, **b** SEM image of a CP crystallite on the scaffold’s surface immersed in SBF for 7 days

The atomic ratio of Ca/P of PHEMA–Gel-1 was demonstrated by EDX analysis (Fig. 8-a). This ratio was about 1.30 which indicated the stoichiometric ratio of another type of CP called octacalcium phosphate (OCP) [70]. Also, the images of the OCP crystallite formed on the scaffold surface could be detected in the SEM of this scaffold (Fig. 8-b).

As was mentioned above, the CP on the surface of the scaffolds is OCP. OCP is considered to be a precursor of apatite formation, since previous researches suggest that OCP requires lower activation energy for nucleation than hydroxyapatite (HA) [71, 72]. The presence of OCP in the mineralization process of bone has also been proven [73–75]. On the other hand, the degradability evaluation of this research showed that the immersed scaffolds in PBS led to a decrease in pH compared to the neutral state in the first week and the pH increased again with the passage of weeks. Since higher pHs lead to more direct HA formations, this issue might be one of the explanations for OCP formation after 7 days soaking in SBF [73, 76].

For the aim of investigating the biomineralization of an electrospun scaffold composed of PCL and OCP, Heydari et al. soaked the samples in SBF for 9 days. The results demonstrated that the addition of OCP significantly enhanced the potential of apatite formation on the scaffold surface [77]. Recently in a research, the bioactivity of a novel scaffold comprised of β-tricalcium phosphate (β-TCP) and OCP have been evaluated by immersing the scaffolds in SBF for 28 days. The authors indicated that OCP had stronger biological activity in comparison with β-TCP since the compactness and uniformity of HA deposition on the scaffolds surface could be improved with the increase in OCP [78].

OCP has appeared beyond expectations in bone regeneration studies. Kamakua et al. examined the potential of bone regeneration for a scaffold composed of collagen and OCP. After 8 weeks of implantation in rat crania, radiographic and histological investigations exhibited significant bone nucleation in the critical-sized bone defects as well as improvement in the percentage of new bone formation compared to the collagen scaffold [79]. In another study, the same authors, evaluated the bone regeneration of collagen/OCP in a clinical trial with two patients who had radicular cysts. After 3 and 6 months of implantation, radiographic results showed efficient healing with a meaningful increase in computed topography. Interestingly, no infection or allergic reactions were observed in the entire period [80]. Bone regeneration capability of OCP has also been proved by histological evaluations of OCP 3-dimensional (3D) printed scaffolds which were implanted in rabbit’s cranial. The scaffolds reduced the diameter of the bone defect 2.5 times in a region where natural bone regeneration was too inefficient [81].

**Fig. 9:**
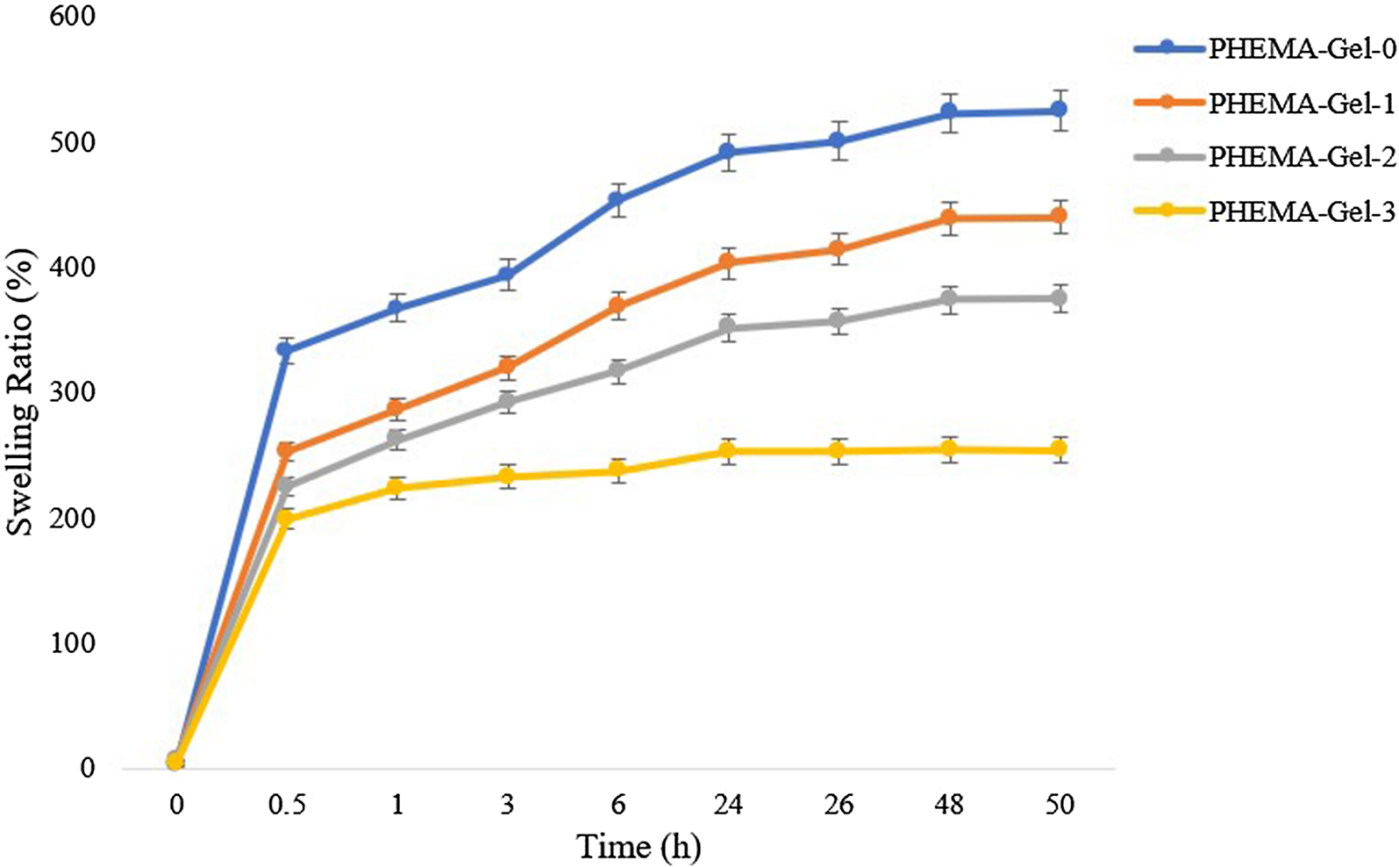
Swelling ratio of the prepared scaffold in PBS (pH = 7.4)

The swelling ratio of a scaffold plays a key role in interacting with cells. On the other hand, it has a significant impact on the rate of scaffold degradation [82]. Figure 9 demonstrates the swelling ratio of the scaffolds after placing in PBS (pH 7.4). As could be observed, in the first 0.5 h, the scaffolds began to absorb water rapidly and the water absorption decreased by increasing the concentration of GO in the structure; given that the water uptake of PHEMA–Gel-0, PHEMA–Gel-1, PHEMA–Gel-2, and PHEMA–Gel-3 were 332, 251, 224 and 198%, respectively. Gradually, at specific time points of 1, 3, 6, 24, and 26 h, the water absorption of the scaffolds increased with maintaining the mentioned trend and reached the equilibrium at 48 h. The PHEMA–Gel-3 reached the equilibrium earlier than the other scaffolds at 24 h (with a final swelling ratio of 254%). The final water uptake of PHEMA–Gel-0, PHEMA–Gel-1, and PHEMA–Gel-2 were 522, 438, and 373%, respectively.

Although it was expected to obtain the reverse trend of swelling ratio compared to the water contact angle as a result of GO addition based on the previous literatures (the less water contact angle resulted in the more swelling ratio), similar pattern was observed for these parameters (except for PHEMA–Gel-3) [83]. The reason could be the effect of the presence of GO on reducing the pore size and hardening of the scaffold. Obviously, as the structure becomes harder and the porosity decreases, water hardly enters the structure. Based on the results, it could be claimed that the stability and maintaining the integrity of the scaffold in the fluid environment inside the body could be improved by increasing the concentration of GO [84–86].

**Fig. 10:**
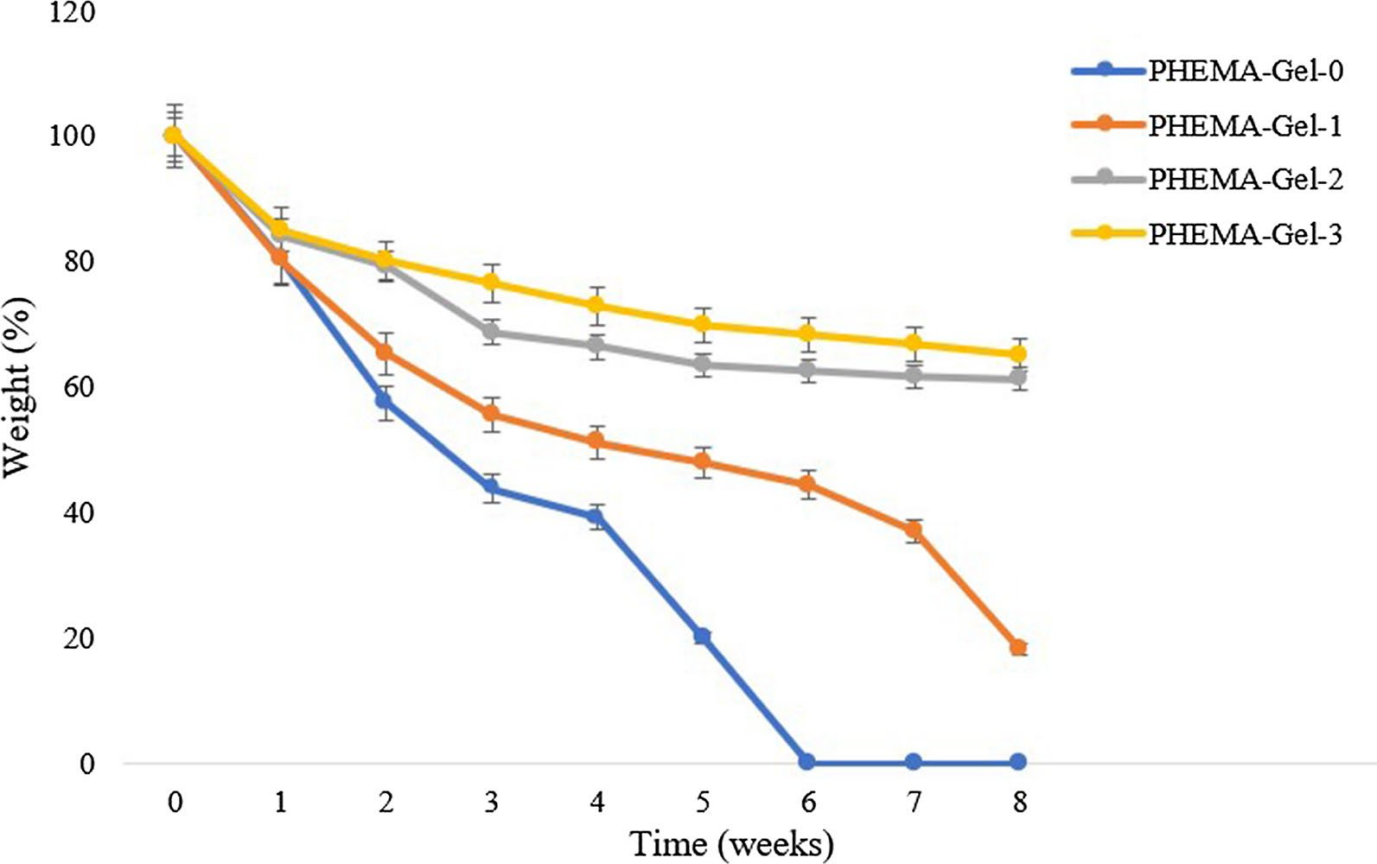
Degradation rate of the prepared scaffold in PBS (pH = 7.4)

The degradation rate has an undeniable effect on the final fate of the scaffold in the body and the repair of damaged tissue [87]. The weight loss percentage of the scaffolds after 8 weeks immersing in PBS (pH 7.4) at 1-week time points is shown in Fig. 10. As can be observed, PHEMA–Gel-0 was rapidly degraded in the first 4 weeks, lost almost 60% of its weight at the end of the fourth week, and completely lost its structure after 6 weeks. PHEMA–Gel-1 had a more relative stability; 49% of its initial weight was lost after 4 weeks and by the end of the 8^th^ week, about 18% of the initial weight remained. However, PHEMA–Gel-2 and PHEMA–Gel-3 maintained their stability and after 8 weeks, they lost only 39% and 35% of their weight, respectively. Since according to the revealed data, the bone repair process will take about 6–8 weeks, the degradation rate of the PHEMA–Gel-2 and PHEMA–Gel-3 scaffolds appears to be commensurate with the mentioned time, because a weight loss of about 40% in 8 weeks maintains the integrity and the essential strength of the structure properly. Besides, it provides the space needed for bone repair and facilitates the process of new bone formation [88]. Therefore, it can be inferred that increasing of GO concentration reduces the degradation rate and supports the integrity of the scaffold structure. This is also in line with the results obtained for the swelling ratio; obviously, the higher water absorption, accelerates the hydrolysis degradation process. The output of the degradation assay is in compliance with the previous researches [84].

**Fig. 11:**
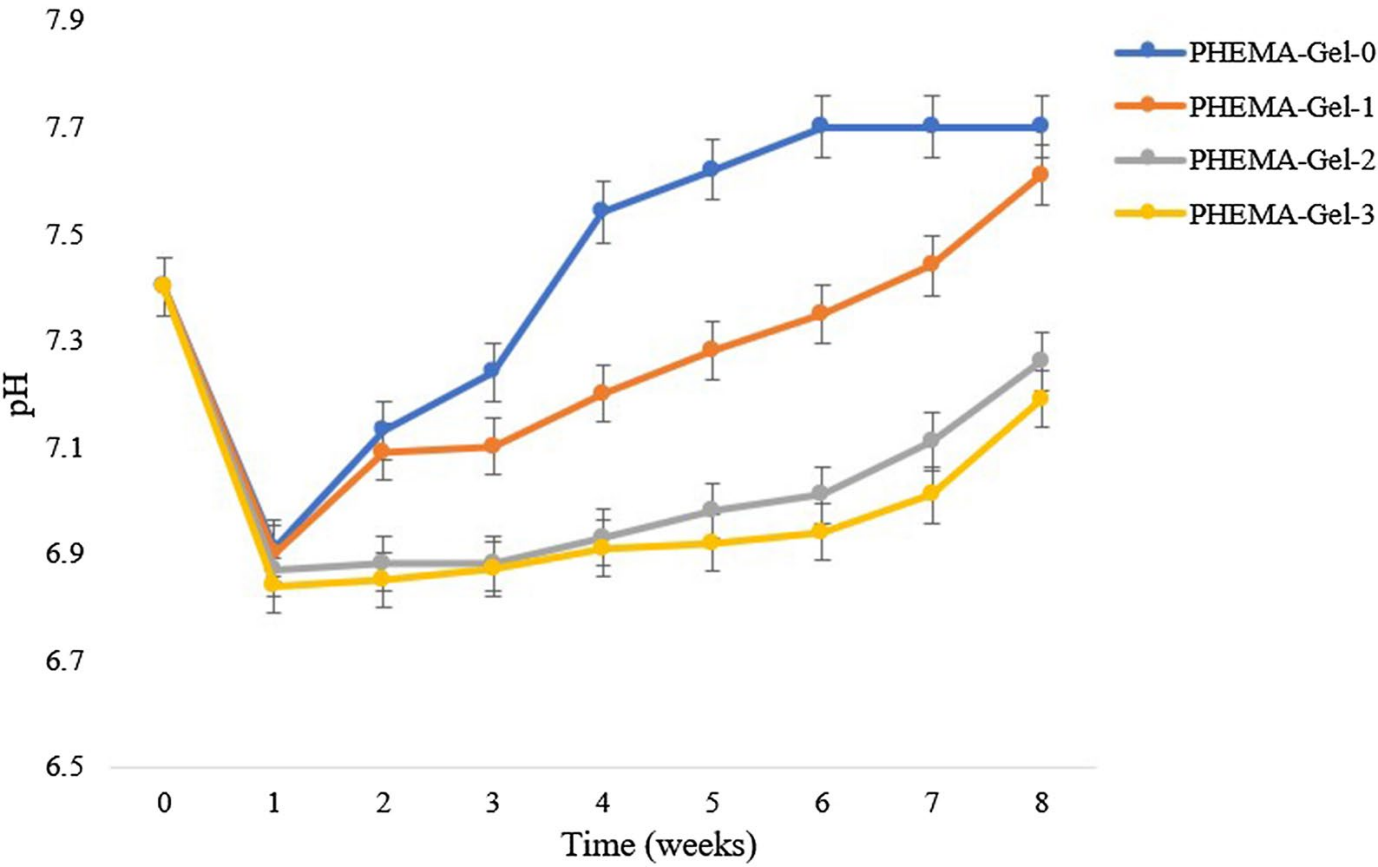
pH of the solution after immersing the prepared scaffold in PBS (primitive pH = 7.4)

For the aim of evaluating the pH of the scaffolds by-products, during the degradation assay and after the scaffolds were removed, the pH of the PBS solutions was measured (Fig. 11). Based on the diagram, it could be stated that the pH decreased compared to the neutral state in the first week and the more GO concentration led to more pH decrease. These pH values increased and became more alkaline by the time passage regarding the noted GO concentration-based trend. The reason behind the decrease in the PH in the first week was probably the release of the particles comprises acidic functional groups such as carboxyl, carbonyl, and hydroxyl [89]. According to the FTIR results, PHEMA contains carbonyl groups and GO contains carbonyl and carboxyl groups. Moreover, the hydroxyl group could be attributed to PHEMA, Gel, and GO. Consequently, the release of the other non-acidic functional groups led to the increase in the PH in the next 7 weeks [90]. Furthermore, more GO concentration resulted in more acidic PH due to the GO acidic functional groups.

### Cellular evaluations

**Fig. 12:**
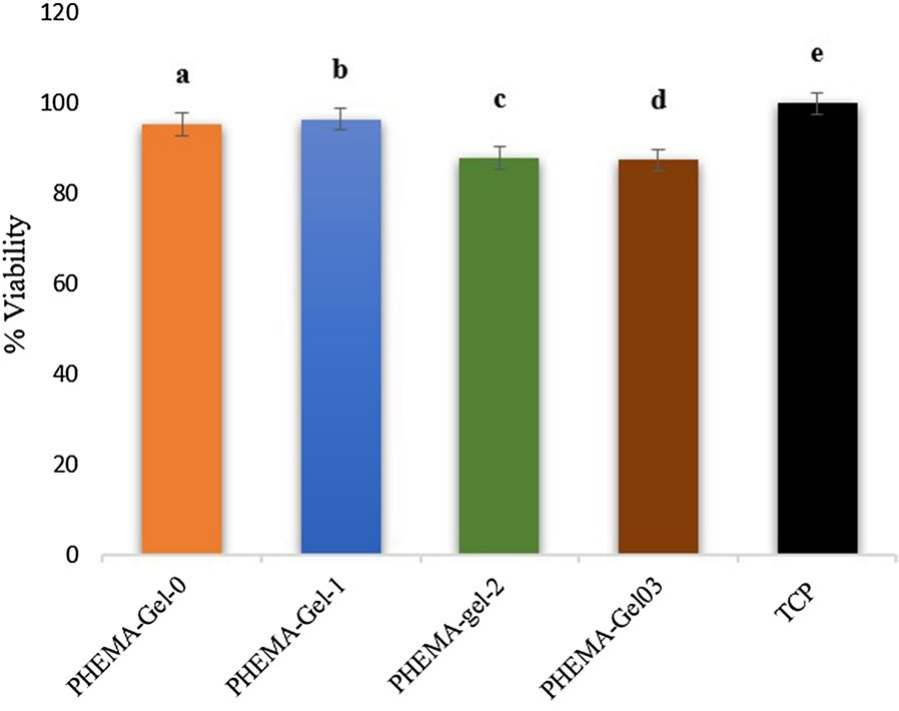
MTT assay results for evaluating the viability of the hBM-MSCs cultured on the scaffolds after 48 h, c and d: significant difference compared to a, b, and e (*p* value < 0.05)

The MTT evaluation was performed to assess hBM-MSCs viability, 48 h after the culture (Fig. 12). According to this chart, cells viability on TCP (control sample) was not significantly different from PHEMA–Gel-0 and PHEMA–Gel-1. PHEMA–Gel-2 and PHEMA–Gel-3 scaffolds presented relatively less viability than TCP, PHEMA–Gel-0, and PHEMA–Gel-1 (*p* value < 0.05). The results of MTT assay indicated the cytocompatibility and non-toxicity of the PHEMA–Gel–GO scaffolds which is in compliance with the recent investigations [23, 57].

**Fig. 13:**
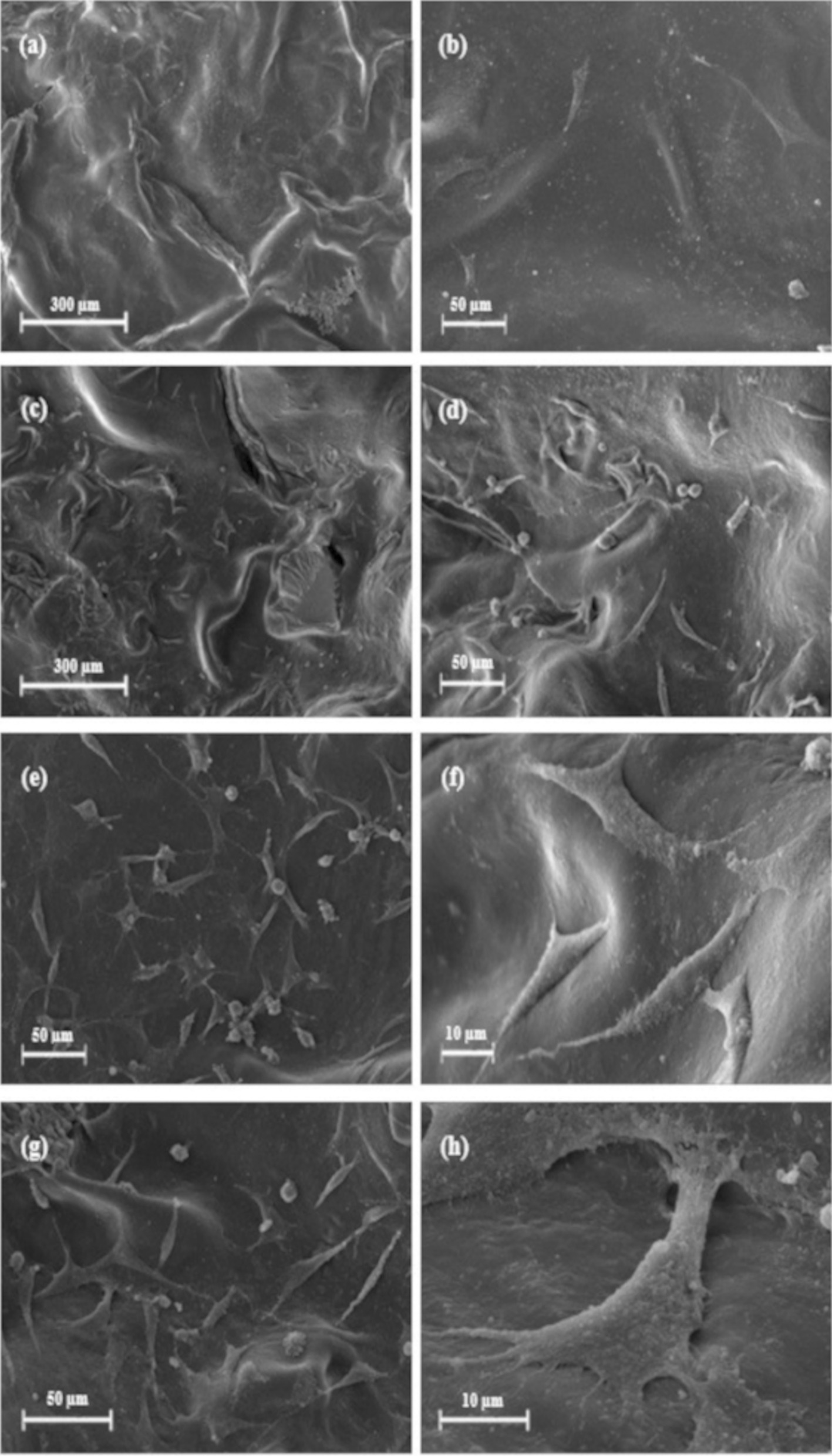
SEM images of the hBM-MSCs cultured on the scaffolds after 48 h with different magnitudes, **a**, **b** PHEMA–Gel-0, **c**, **d** PHEMA–Gel-1, **e**, **f** PHEMA–Gel-2, and **g**, **h** PHEMA–Gel-3

Figure 13 shows the SEM images of the seeded hBM-MSCs on the scaffolds after 48 h. The adhesion of cells on PHEMA–Gel-0 (Fig. 13a, b) is rarely observed in a scattered manner. However, with the addition of GO, not only the number of cells located on the surface of the scaffolds increased, but also the scaffolds containing GO seemed to have a much better interaction with the cells and that is due to the fact that the cells have expanded their appendages and become elongated, which have been amplified by the increase in GO concentration. This result indicates that the presence of GO can facilitate and support the cell adhesion and growth by providing a favorable substrate [8].

**Fig. 14:**
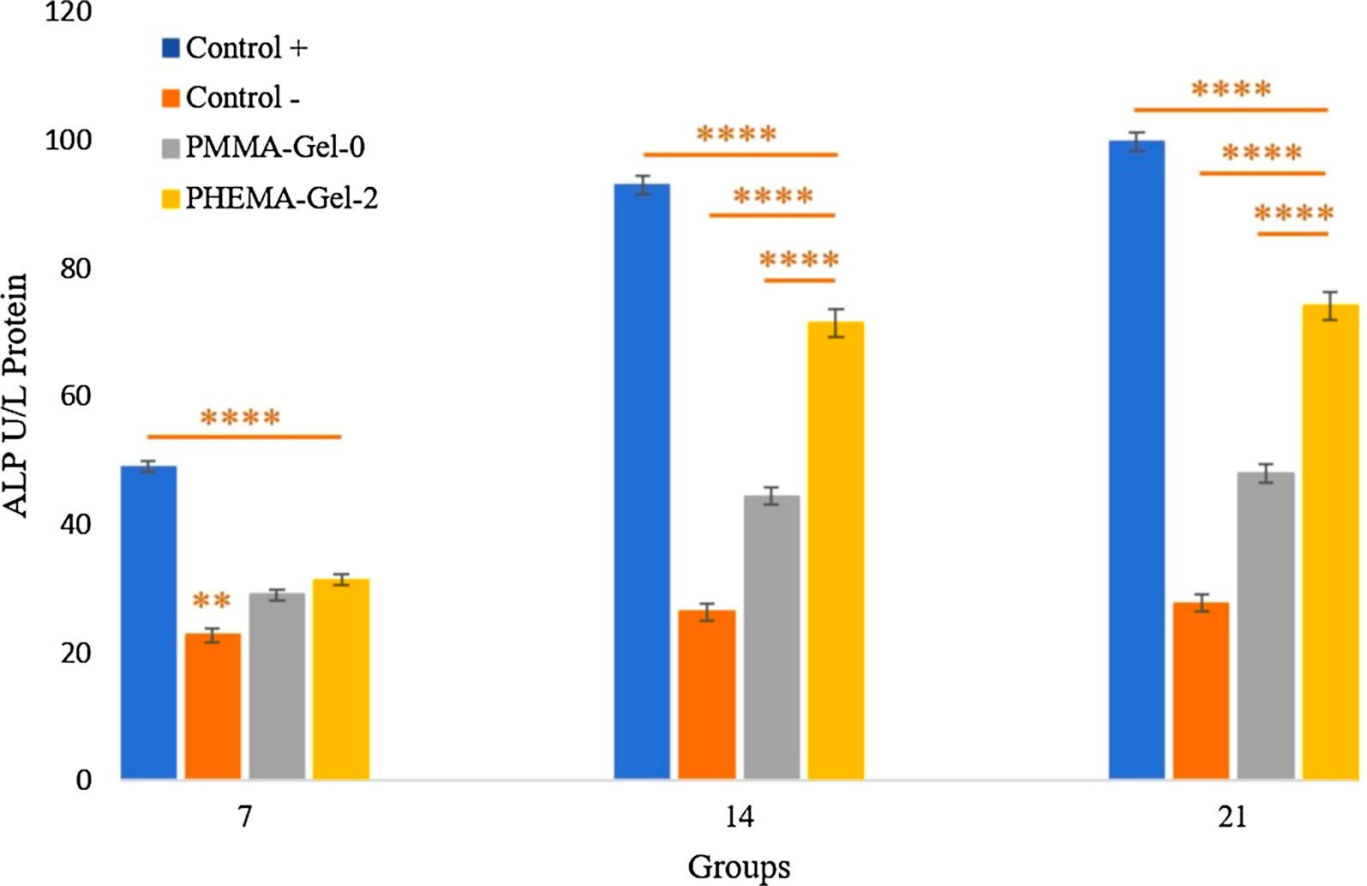
ALP activity of the hBM-MSCs cultured on control groups and scaffolds after 7, 14, and 21 days, (****p values < 0.0001 compared to other groups, ** = significant difference compared to PHEMA–Gel-2 (*p* value < 0.01)

Figure 14 shows the ALP expression of the prepared scaffolds after 7, 14, and 21 days. It should be noted that the PHEMA–Gel-2 was selected as the representative sample for this analysis. This sample was selected due to its desirable structural and cellular characterizations results obtained on previous characterizations. As can be observed, at day 7, PHEMA–Gel-2 scaffold demonstrated a significant higher expression than the negative control (without osteogenic differentiation medium). At day 14, remarkable increase could be seen for PHEMA–Gel-0 and PHEMA–Gel-2 scaffold compared to the negative control. Moreover, the PHEMA–Gel-2 scaffold had a greater osteogenic differentiation than the PHEMA–Gel-0 scaffold. The same trend was also maintained at day 21 and the ultimate ALP expression for PHEMA–Gel-2 scaffold reached approximately 70% of the positive group (with osteogenic differentiation medium).

The reason behind the improvement of the ALP activity by GO addition could be attributed to the stiff augmentation of GO as aforementioned. According to the earlier researches, the structural stiffness caused by the presence of GO could alter the cellular morphology through creating mechanical stimulation and impacting on the cytoskeleton tension [91]. This occurrence would increase the possibility of differentiation to bone cell lines since the prepared synthetic structure can better mimic the ECM of natural bone tissue [92, 93]. Moreover, this biophysical factor as a mechanotransduction cascade initiator, performs a key role on controlling the intracellular pathways and accelerating the osteogenic differentiation of the stem cells by transforming the intra-mechanical forces into biochemical differentiative cues [94–96]. As stated in the previous literatures, GO could regulate the bone morphogenic protein (BMP) and mitogen-activated protein kinase (MAPK) signaling pathways which are effective for osteogenic differentiation [91, 97, 98]. On the other hand, it has been reported that the hydroxyl functional group of GO can enhance the hydrogen bonding between the scaffold surface and the existing protein which eventually causes more cellular affinity to the surface. It was revealed in a study that GO could affect the induction of prostaglandin E2 (PGE2) secretion and upregulation of transforming growth factor beta 1 (TGF-β1). PGE2 and TGF-β1 are both important factors for osteoblastic differentiation by the increase in ALP activity [99]. Thus, it could be claimed that the prepared PHEMA–Gel-2 scaffold has an acceptable capability for osteogenic differentiation.

## Conclusion

According to the findings, the inclusion of GO rendered the scaffolds suitable for use as a load-bearing tissue in bone. The electroactivity and bioactivity of the scaffolds caused by GO were also considerable characteristics as they led to the optimum mimicking of the bone electrophysiologic environment. The increase in GO concentration approximated the degradation rate of the scaffolds to the bone repairing process. The osteogenic capability of the scaffolds was further enhanced by the addition of GO, which was approved by ALP expression. Considering the obtained desirable structural properties and the proper cellular interactions, it is suggested to assess the efficiency of the prepared composition in vivo. More experiments are also required to investigate the potential of the fabricated structure for possible clinical application. Also, computer-aided design (CAD) methods could be utilized to optimize the concentrations of the individual components as well as the structural indicators such as dimensions and pore size and shape.

## Data Availability

All data generated or analyzed during this study are included in this published article (and its supplementary information files).

## Abbreviations

GO: Graphene oxide
PHEMA: Poly(2-hydroxyethyl methacrylate)
FTIR: Fourier-transform infrared spectroscopy
XRD: X-ray diffraction assay
SEM: Scanning electron microscopy
MTT: 3-(4,5-Dimethylthiazol-2-yl)-2,5-diphenyl-2H-tetrazolium bromide
hBM-MSCs: Human bone marrow‐derived mesenchymal stem cells
ALP: Alkaline phosphatase
PLA: Polylactic acid
PGA: Polyglycolic acid
PCL: Polycaprolactone
Gel: Gelatin
RGD: Arg–Gly–Asp
DMF: Dimethylformamide
EDC: 1-Ethyl-3-(3-dimethylaminopropyl) carbodiimide
NHS: *N*-Hydroxysuccinimide
DI: Deionized
SBF: Simulated body fluid
EDX: Energy-dispersive X-ray analyzer
PBS: Phosphate-buffered saline
DMEMF12: Dulbecco’s modified Eagle medium F12
FBS: Fetal bovine serum
UV: Ultraviolet
DMSO: Dimethyl sulfoxide
GA: Glutaraldehyde
pNPP: Nitrophenylphosphate
SDs: ± Standard deviation
CP: Calcium phosphate
OCP: Octacalcium phosphate
HA: Hydroxyapatite
β-TCP: β-Tricalcium phosphate
3D: 3-Dimensional
ECM: Extracellular matrix
BMP: Bone morphogenic protein
MAPK: Mitogen-activated protein kinase
PGE2: Prostaglandin E2
TGF-β1: Transforming growth factor beta 1
